# Rings in Clinical Trials and Drugs: Present and Future

**DOI:** 10.1021/acs.jmedchem.2c00473

**Published:** 2022-06-22

**Authors:** Jonathan Shearer, Jose L. Castro, Alastair D. G. Lawson, Malcolm MacCoss, Richard D. Taylor

**Affiliations:** † UCB, 216 Bath Road, SloughSL1 3WE, United Kingdom; ‡ Bohicket Pharma Consulting Limited Liability Company, 2556 Seabrook Island Road, Seabrook Island, South Carolina29455, United States

## Abstract

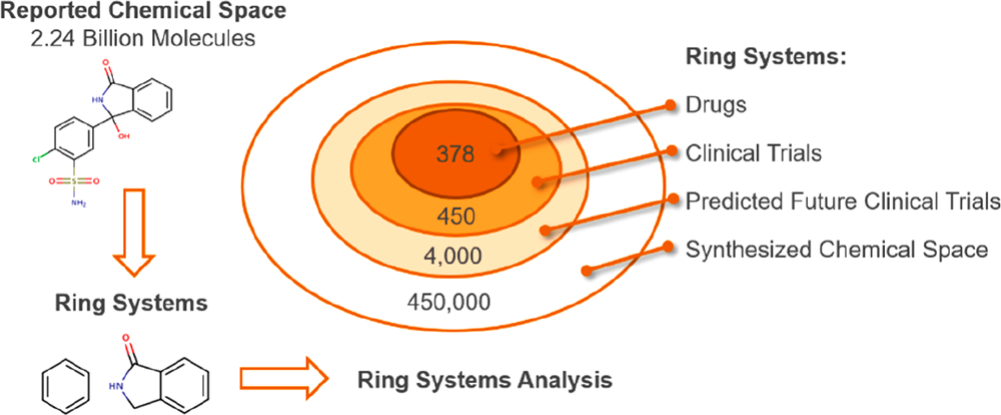

We present a comprehensive analysis of all ring systems (both heterocyclic and nonheterocyclic) in clinical trial compounds and FDA-approved drugs. We show 67% of small molecules in clinical trials comprise only ring systems found in marketed drugs, which mirrors previously published findings for newly approved drugs. We also show there are approximately 450 000 unique ring systems derived from 2.24 billion molecules currently available in synthesized chemical space, and molecules in clinical trials utilize only 0.1% of this available pool. Moreover, there are fewer ring systems in drugs compared with those in clinical trials, but this is balanced by the drug ring systems being reused more often. Furthermore, systematic changes of up to two atoms on existing drug and clinical trial ring systems give a set of 3902 future clinical trial ring systems, which are predicted to cover approximately 50% of the novel ring systems entering clinical trials.

## Introduction

Drug-like chemical space is a phrase ubiquitous in drug discovery. It is of fundamental importance in small molecule drug discovery and impacts all stages of the design cycle from screening library design, through to reagent selection, hit to lead, and lead optimization. However, for such an extensively used description, which impacts most decisions in drug discovery, there is no accepted gold standard measure to delineate “drug-like chemical space” which is universally applicable and unambiguously encompasses, without exception, any molecule that is drug-like.

The term drug-like is fraught with ambiguity; for example, it could mean (i) an exact substructure found in a drug, (ii) a closely related substructure, (iii) a closely related full molecule structure, or (iv) a molecule that has similar properties to known drugs. How to calculate what is close or similar can be achieved using a plethora of computational techniques ranging from 1-dimensional (1D) properties such as calculated logP (clogP), polar surface area (PSA), and hydrogen-bonding groups (H–B donors or acceptors) to 2-dimensional (2D) metrics such as fingerprint similarity, the presence or absence of functional groups, up to 3-dimensional (3D) metrics such as shape or molecular electrostatic potentials.^1,2^ A further complication of drug likeness is the nonbinary nature of the description where we consider the drug likeness not to be true or false but defined on a continuum with a relative probability.^3^

Many early attempts at estimating drug-like space use weighted combinations of whole molecule properties, often referred to as 1D properties, such as molecular weight or polar surface area.^4−6^ In parallel, 2D and 3D descriptors have been used to identify molecules that are in drug-like space.^7,8^ Similar analysis to drug-like space has been applied to molecules in clinical trials showing in some cases that molecules in clinical trials have significantly different property space compared with molecules that have transitioned successfully to a marketed drug.^9^

## Size of Drug-Like Space

**Figure 1:**
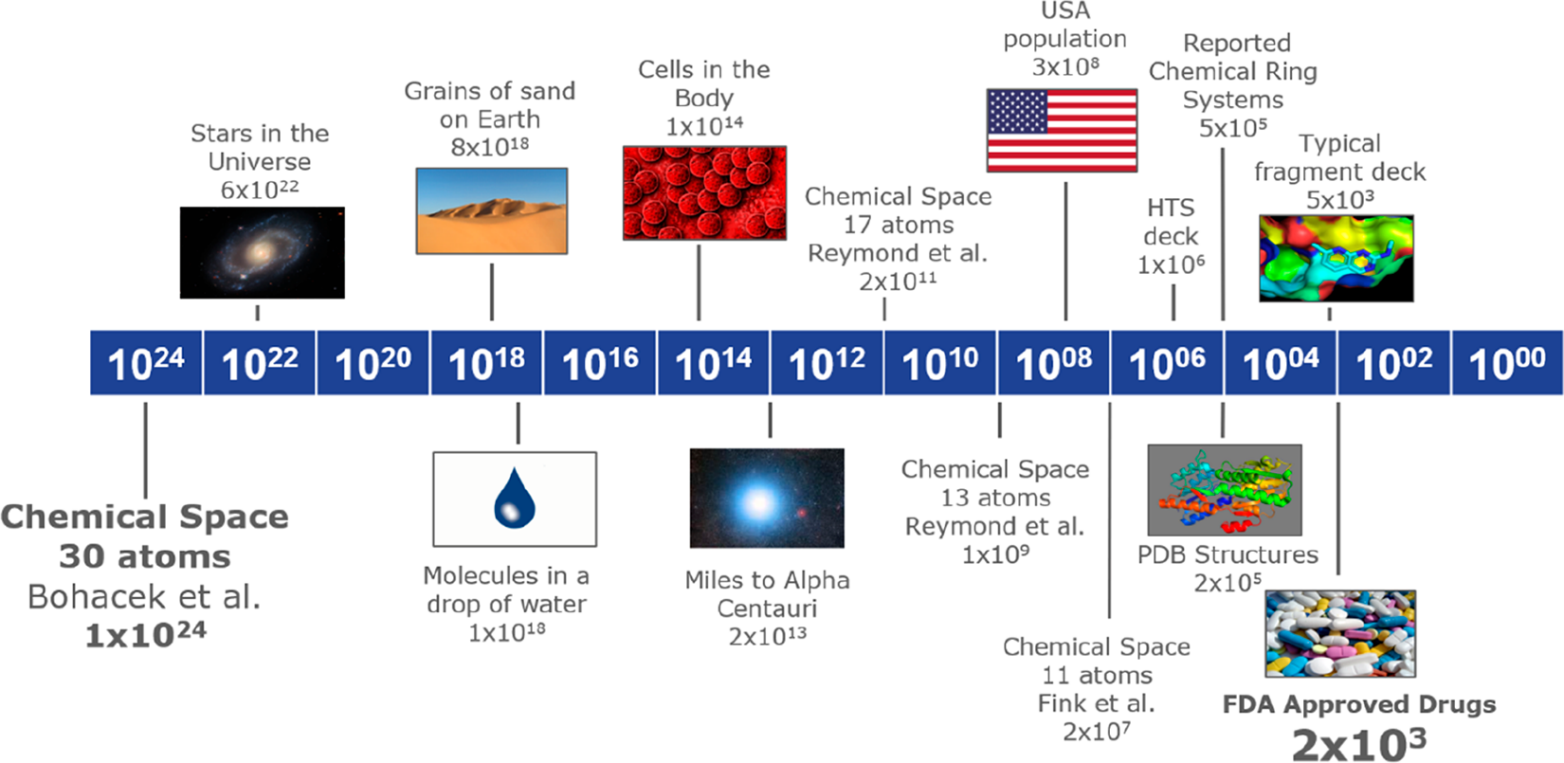
Summary of predicted size of chemical space and key comparisons. Some images were sourced from third parties: “Stars in universe”, permission given by NASA;^17^ “Grains of sand on Earth”, permission given by FreeImages;^18^ “FDA Approved Drugs”, permission given by FreeImages;^19^ “Miles to Alpha Centauri”, permission given by European Southern Observatory (ESO), Davide De Martin.^20^

The success of combining 1D properties is in part due to the ease and speed of calculations, enabling rapid assessment of libraries of molecules, and these approaches clearly had a significant impact on the field of drug discovery. Notable examples are the Lipinski ubiquitous “Rule of 5” (Ro5),^10^ the work of Veber^11^ based around PSA, and many others including the “GSK 4/400” for lead-like molecules and “Rule of 3” for fragment molecules.^12−14^ However, there are widely accepted drawbacks, namely, the problem of successful drugs that lie outside of these models, and as a result many seasoned practitioners in small molecule drug discovery would typically find utility of these methods as a probabilistic guide rather than a binary cutoff. Another less widely discussed difficulty centers around the size of drug-like chemical space. Even using guides such as the Ro5 the enormity of drug-like space within these guides is still problematic. This is summarized in Figure 1, which highlights the size of chemical space for drug discovery along with related data to give context to the data size. Moreover, we will show an estimate of currently available chemical ring systems using our previously described ring system definitions^15,16^ is approximately 5 × 10^5^ ring systems (excluding macrocycles), which is also included in Figure 1.

Although we have the capabilities to experimentally screen multimillion compound libraries and virtual screens of billions of molecules have been reported,^21^ the size of the predicted chemical space is still beyond the reach for routine screening and even virtual enumeration. Some examples of current state of the art libraries include Enamine Real Space^22^ (2 × 10^10^), GalaXi/Wuxi LabNetwork (2 × 10^9^), and GDB 17 (2 × 10^11^).^23^ The obvious question then is how can we reduce this size further with a measure that is unambiguous, clearly defined, and easy to calculate to identify pockets of useful molecules in a space that is larger than the number of stars in the universe.^24^ As previously described,^15^ we chose substructure analysis based around ring systems and frameworks rather than simple whole molecule properties or similarity metrics based on 2D or 3D descriptors to explore drug-like chemical space.

## History of Scaffold Analysis

Ring systems are highly influential in determining the shape, electrostatics, and often bioactivity of compounds. The concept of such “privileged” or bioactive scaffolds has been widely explored in the drug discovery field.^25−28^ There are many ways to define a molecular scaffold, but arguably, the most widely used way would be the Bemis–Murcko (BM) scaffold.^29^ The BM scaffold is obtained by removing all terminal acyclic groups from the ring systems and frameworks. A further simplification of the BM scaffold can be obtained by ignoring atom types and bond orders in the graph to give the cyclic skeleton (CSK). The CSK allows for a more basic comparison of the underlying shape of each scaffold. BM and CSK scaffolds have been shown to be powerful tools for analyzing the diversity within any compound collection. In the original paper by Bemis and Murcko it was found that one-half of all drug molecules could be represented with 32 frameworks. A more recent paper by Lipkus et al.^30^ defined an indicator of the innovation present in a new drug by comparing their scaffolds to those already in existing drugs. In this work, they defined an innovative drug, or “Pioneer”, by whether both the scaffold and the molecular shape had not been observed in a previous drug molecule. Their analysis was carried out on all approved drugs over the last 80 years, and they showed that the percentage of new scaffolds combined with molecular shape has increased over time, where the scaffolds and shapes are defined using their reduced representations.

A key use of scaffold generation has been to identify bioactive ring systems or frameworks. Techniques such as scaffold trees,^31,32^ hierarchical scaffold clustering,^33^ and match molecular pairs analysis^34^ (MMPAs) are commonly used to identify structure–activity relationships (SAR) across a compound collection and thus identify potential scaffold hops.^35^ Other work has combined the use of quantitative structure–activity relationship (QSAR) models^36,37^ of full molecules and a molecule generator to identify bioactive scaffolds.

Visini et al.^38^ generated a database of all possible rings (1–4 rings, <30 atoms), resulting in around 1 million virtual ring systems, 98.6% of which had not been observed in any publicly available compound collections (ZINC,^39^ PubChem,^40^ ChEMBL,^41^ and Reaxys^42^). It should be noted that these rings were not filtered by whether they were drug-like or synthetically feasible; thus, it is not clear how many of these rings are biologically relevant. An alternative analysis by Pitt et al.^43^ estimated that there could be over 3000 ring systems that have not been reported in the literature but are synthetically tractable. Another study enumerated all possible fused rings up to 3 rings to give around 570 000 virtual ring systems.^44^ These 570 000 ring systems were then cross referenced with those available in the Synthetically Accessible Virtual Inventory^45^ (SAVI, 1.75 billion molecules) to identify 39 036 ring systems.^46^ In this study, data from ChEMBL was used to identify bioactive ring systems that were selective against certain target classes or generally bioactive. The chemical space of these ring systems was visualized by applying PCA on key scaffold descriptors; the scaffold descriptors are described in previous work.^47^ The resulting analysis showed that bioactive scaffolds were spread across chemical space but with local regions of high density. It was hypothesized that the regions of high density could be used to identify future bioactive scaffolds. The identification of such bioactive islands in chemical space has been explored in the literature for both scaffolds^44,46^ and drug-like compounds.^23,48^ Analysis of kinase inhibitors by Zhao and Caflisch^48^ demonstrated that a huge fraction of synthesizable kinase-relevant chemical space has been completely unexplored. By identifying biologically relevant areas of chemical space, the goal is to reduce the effective chemical space for hit discovery and increase hit quality while still generating novel chemical matter.

We previously^15,16^ chose ring systems and frameworks to analyze drugs and drug-like space based on the seminal work of Murcko et al.,^29^ whereby we performed both an exhaustive and a recursive breakdown of molecules and systematically analyzed both the individual components and combinations of scaffolds. It was found that each year 70% of drugs are comprised of only ring systems found in previously marketed drugs. Most of the remaining drugs contain only one newly utilized ring system that has not been seen in marketed drugs. This observation has held true year on year for the last 30 years. This gives rise to the question facing seasoned practitioners of drug discovery: how much chemical novelty is required for a patentable and effective drug, and how is this novelty achieved? Since 70% of new drugs coming onto the market each year only contain ring systems from previously patented drug molecules then most new drugs achieve novel patent space through either the utilization of new growth vectors and/or novel combinations of growth vectors or simply novel combinations of drug ring systems. This suggests novel ring systems are not a prerequisite for new patent positions or to tackle new drug targets since we have also shown previously that known drug rings have been applied across different therapeutic targets and therapeutic areas.^15^ In this work, we address the question of novelty and how it applies to new candidates by studying molecules before they make it as drugs, i.e., those in different clinical phases.

## Clinical Trial Ring Systems

Assessing compounds that are earlier in the drug discovery cycle that have not yet made it to market and are currently in clinical trials can give a further insight into the successful design of future drug molecules. This is the focus of this study, whereby we analyzed the chemical novelty in clinical trials using an extended methodology that we previously applied to drug molecules to answer the following questions.

(1) Is the amount of molecular novelty (where novelty is assessed by new chemical ring systems) in drugs reflected in clinical trials or is there an attrition in clinical trials?
(2) Is the amount of novelty different across the different clinical phases?
(3) How important are new chemical ring systems to justify clinical investment and overall clinical success, and how do we use these data?
(4) Can we use the novelty from clinical trials to predict future drug ring systems and prioritize new areas of available chemical space?

## Fragmentation Methodology and Classifications

**Figure 2:**
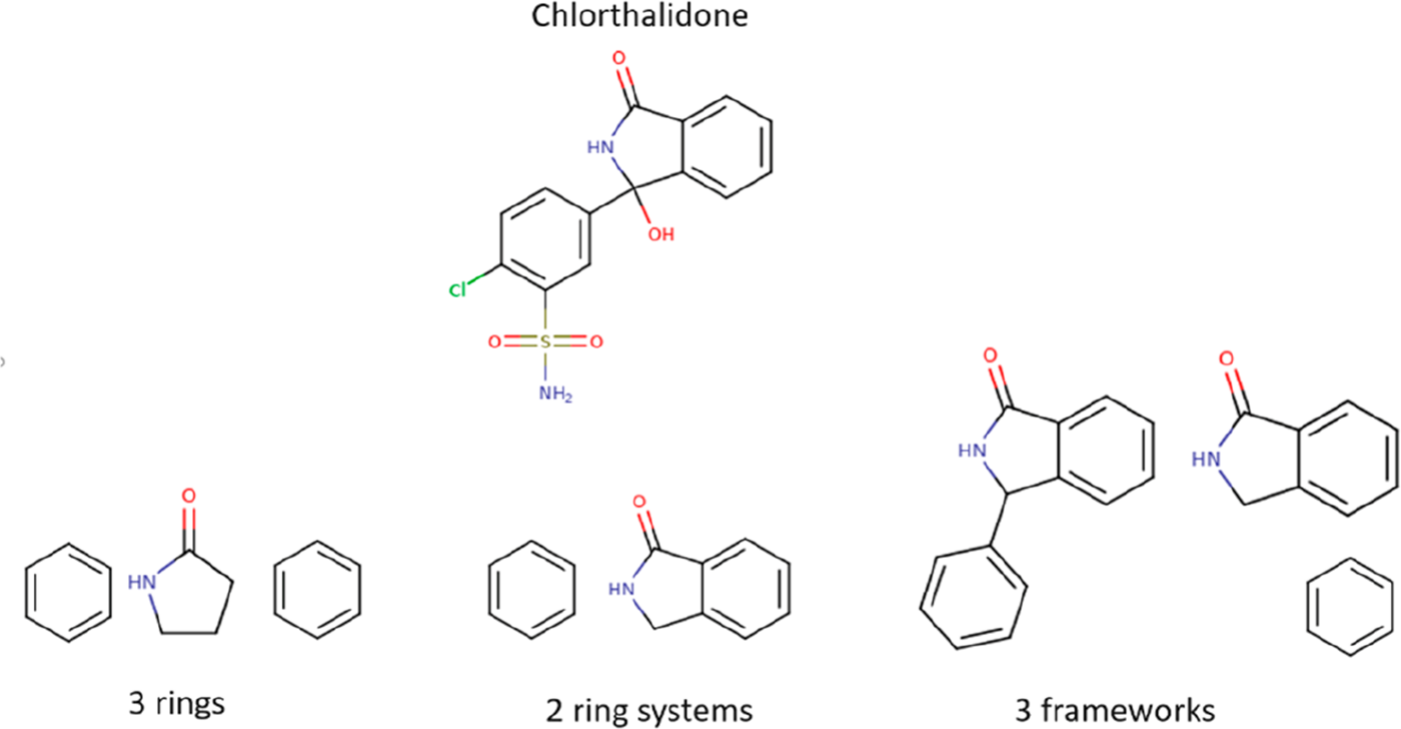
Example of rings, ring systems, and frameworks for Chlorthalidone.

To answer these questions, we used the same methodology as described in previous work^15,16^ to deconstruct molecules, whereby the fragmentation algorithm recursively breaks each molecule into ring systems and frameworks with exocyclic double bonds retained while recording the growth vectors of each ring system from the frameworks (see Figure 2). We applied this algorithm to a snapshot of molecules in Phase 1, 2, and 3 clinical trials from January 2020 and our updated drug data set reported in the US FDA Orange Book up to January 2020. This gave us an updated ring system from drugs and/or clinical trials and associated frequencies and growth vectors. The fragmentation workflow was implemented using a combination of RDKit^49^ and Pipeline Pilot.^50^

We filtered the sets so that they contain less than 10 bonds in a ring, no metal-containing molecules, and a molecular weight less than 1000. The molecular weight cutoff was chosen to capture larger small molecules that fall outside of Ro5 while removing excessively large molecules. Previous studies^51,52^ have shown that applying a hard cutoff on weight in line with Ro5 can lead to the loss of a number oral drugs or clinical candidates for difficult target classes that may contain innovative scaffolds of interest to our analysis. We also ensured that the molecules in the different phases must not be present in higher phases or drugs.

Using the sets of different ring systems from molecules in clinical trials and drug molecules, we propose a new classification system based on the ring systems (or scaffolds) present in a molecule. This classification is to give a simple and clear description of a molecule to show the degree of chemical novelty and the importance of reuse of existing scaffolds vs new scaffolds. This classification is given in Table 1, and as a point of clarification, a new ring system is one that has not been used previously in any other drug that has made it to market. For this analysis, we have not focused on molecules that do not contain any ring systems (Class 5), which typically accounts for less than 10% of drugs.

**Table 1:** Molecule Classification System Based on Historical Context of Ring Systems

molecule class	description of ring systems contained in the molecule
Class 1	only known drug ring systems, combined in a novel way
Class 2	known drug ring systems combined with only 1 new ring system
Class 3	known drug ring systems combined with more than 1 new ring system
Class 4a	only one ring system in the whole molecule, and that ring system is new
Class 4b	only new ring systems where the total number of ring systems is more than 1
Class 5	no ring systems present

Using the classification from Table 1, our previous work showed 70% of drugs each year come under the Class 1 molecules; the remaining drugs are formed predominantly from Class 2 molecules. Even with a significant representation of Class 2, Class 3, and Class 4 in commercially available molecules and literature-reported molecules, Class 1 molecules are still the most successful and dominant class in marketed drugs and have been, year on year, for the last 30 years. The benefit of Class 1 is that we can map out the space and cleanly define this data set, which we have previously enumerated, combined, and analyzed.^16^ Class 2, Class 3, and Class 4 can be estimated, but full and complete enumeration is a significant computational undertaking. Using these classifications, we analyzed clinical trial compounds to see whether this distribution is the same for molecules in U.S. clinical trials.

## Classification of Clinical Trial Molecules

The analysis of ring systems present in molecules in the different phases of clinical trials (for January 2020) is shown in Table 2. It can be seen from this analysis that most molecules still fall in the Class 1 bracket with an average of 67% of the compounds across all phases. This mirrors the 70% of Class 1 molecules in drugs. Although there is a slight increase in the different clinical phases, we do not believe this is significant. In terms of novelty in clinical trials, where novelty is assessed by the ring systems and the associated ratio of new vs old, it seems there is little difference between molecules that have made it as a drug and those in clinical trials.

**Table 2:** Classification of Molecules in Different Clinical Phases (January 2020)

clinical trials status	no. of compounds	Class 1(only drug ring systems)	Class 2(drug ring systems and 1 new ring system)	Class 3(drug ring systems and 2+ new ring systems)	Class 4a(single nondrug ring systems)	Class 4b(only 2+ nondrug ring systems)
Phase 1	277	178 (64%)	86 (31%)	4 (1%)	9 (3%)	0 (0%)
Phase 2	525	359 (68%)	123 (23%)	10 (2%)	32 (6%)	1 (<1%)
Phase 3	232	159 (69%)	44 (19%)	8 (3%)	17 (7%)	4 (2%)
all phases	1034	696 (67%)	253 (24%)	22 (2%)	58 (6%)	5 (<1%)

It is an interesting observation that the percentage of molecules containing just drug rings (Class 1) is broadly the same across different clinical trials, and this mirrors new drugs coming onto the market each year. One might have expected there to be more novelty in clinical trials, and this novelty may be reduced through the clinical trials, but this is not the case when novelty is defined by the ring system chemistry.

A conclusion from these observations is that using just drug rings and combining them in novel ways is the principal strategy employed historically to generate most compounds for both new drugs and molecules that make it into clinical trials. Moreover, since on average it takes 9 years for a molecule to pass through clinical trials^53^ to make it to market, this data set will encompass all new drug ring systems for the next 9 years.

## Analysis of Ring Systems in Different Clinical Phases

We have demonstrated a classification of molecules based on ring systems and whether those ring systems have been used previously in drugs. To extend this analysis, we have taken the updated list of drugs and clinical trial molecules and analyzed the complete database of ring systems that are present in these molecules (see Table 3). There are 378 ring systems used in drugs and 450 unique ring systems in clinical trials; 280 (62%) of these clinical trial ring systems have not been used in drugs before. This gives a total of 658 unique ring systems covering all clinical trial molecules and drugs. The fact that there are more ring systems in clinical trials than in drugs demonstrates that there is still a significant investment in new ring systems in drug discovery. However, what is clear is how they are assembled in real molecules is of equal importance when assessing novelty, and new ring systems are typically partnered with known drug ring systems for both clinical trial compounds and drugs. The top 100 ring systems in drugs and top 100 new ring systems in clinical trials are shown in Tables 4 and 5, respectively. The complete lists, ordered by frequency, are available as a pdf download and smiles download from Zenodo (10.5281/zenodo.6556751).

**Table 3:** Classification of Ring Systems in Different Clinical Phases and in Drugs

status	total no. of unique ring systems	new ring systems not in higher phases nor in drugs	ring systems from drugs	ring systems present in higher phases
Phase 1	191	71 (37%)	101 (53%)	19 (10%)
Phase 2	278	131 (47%)	128 (46%)	19 (7%)
Phase 3	169	78 (46%)	91 (54%)	
all phases	450	280 (62%)	170 (38%)	
drugs	378			

**Table 4:**
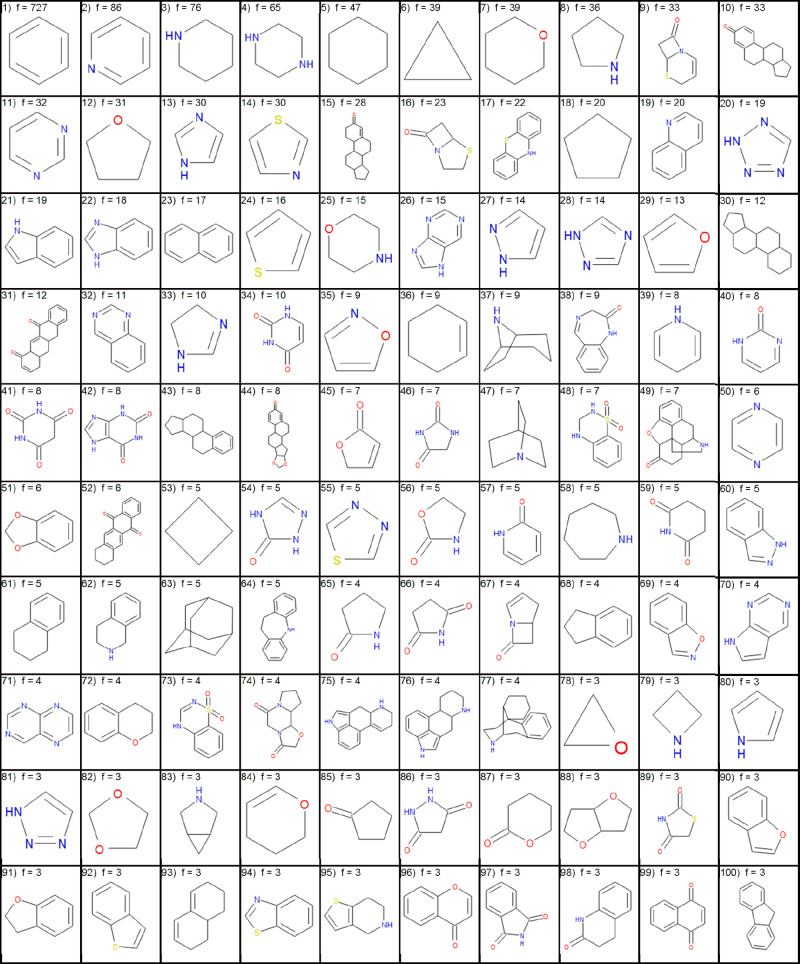
Top 100 Most Frequently Used Ring Systems from Small Molecule Drugs Listed in the FDA Orange Book before January 2020 Sorted by Descending Frequency (f) and Then Ascending Molecular Weight

**Table 5:**
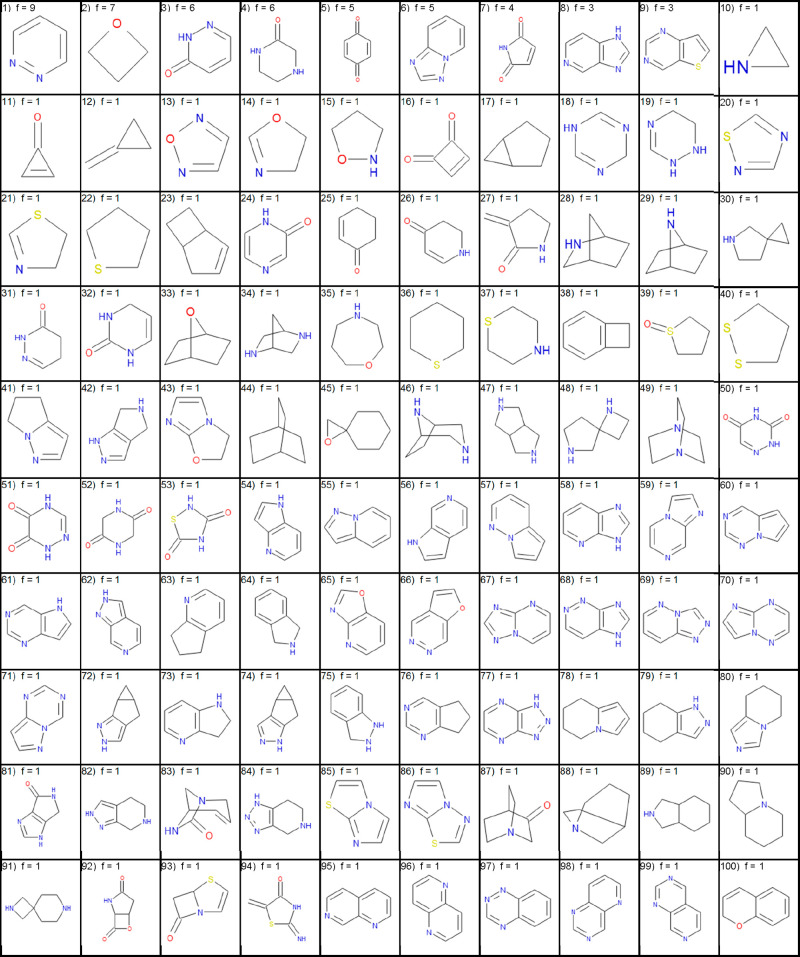
Top 100 Most Frequently Used Ring Systems in U.S. Clinical Trial Compounds in January 2020 That Were Not Present in Drugs Sorted by Descending Frequency (f) and Then Ascending Molecular Weight

Table 3 shows that the sets of ring systems in Phase 2 and Phase 3 have similar distributions between the new ring systems and the ring systems seen in drugs or higher clinical phases. Phase 1 is slightly lower, which could be accounted for by not all structures in Phase 1 being available, and typically structures are not released until Phase 2 or higher. From the pool of ring systems derived from clinical trial molecules, there are more new ring systems being used than ring systems from drugs (62% compared with 38%, respectively). However, the drug ring systems are used in multiple molecules and typically have a higher frequency in clinical trial molecules than the new ring systems. Thus, in the final molecules the drug ring systems dominate through reuse even though it is a smaller pool of ring systems, indicating how important this set is. If 170 drug ring systems are used in clinical trials, this means that 98 drug ring systems are currently not being utilized in clinical trial molecules. From this analysis, several questions arise. Is there a difference in those drug ring systems that are being reused and those that are not? Can we learn anything from those drug ring systems that have not found utility in current clinical trials? Moreover, is there a systematic difference between the properties of ring systems in drugs and those newer ring systems only found in clinical trials?

## Comparison of Properties for Novel Clinical Trial Rings Compared with Drug Rings

To answer the questions regarding the differences between ring systems in clinical trials and drugs we calculated the distributions of ring sizes, the number of nitrogens, oxygens, sulfurs, and combined heteroatoms, as well as the number of sp^3^ centers. Many of these 1d properties are presented as percentages to allow for the comparison of ring systems with disparate sizes. For this analysis, we separated the ring systems into the following categories:

(a) ring systems from drugs,
(b) ring systems only in Phase 3, Phase 2, or Phase 1 (and not in higher phases or drugs),
(c) all ring systems combined from all clinical phases (but not in drugs),
(d) ring systems just from drugs that are not being used in clinical trials.

**Figure 3:**
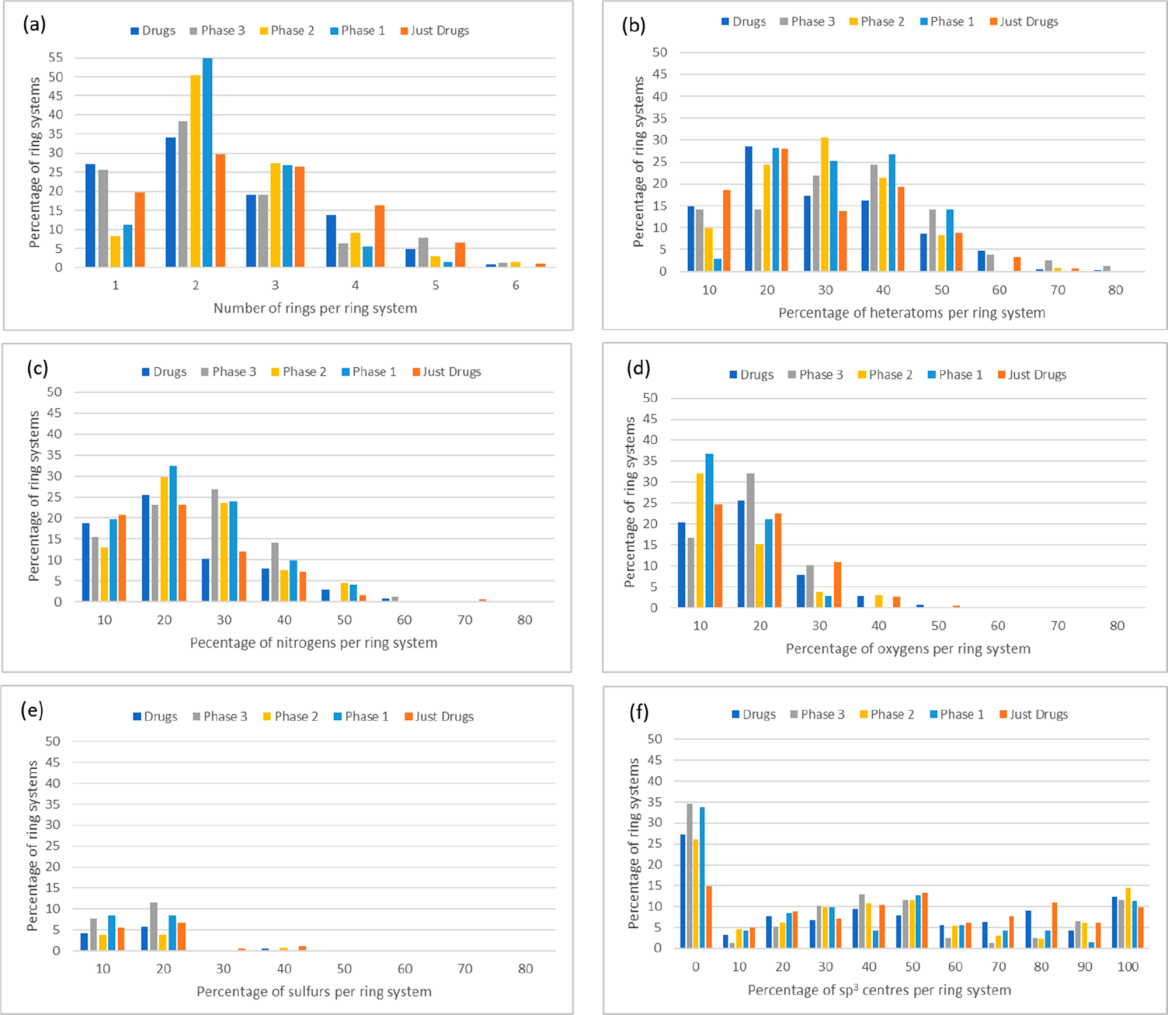
Histogram comparisons of ring systems in drugs, clinical trials, and those exclusively in drugs that are not currently in clinical trials for (a) number of rings per ring system, (b) percentage of heteroatoms per ring system, (c) percentage of nitrogens per ring system, (d) percentage of oxygens per ring system, (e) percentage of sulfurs per ring system, and (f) percentage of sp^3^ centers per ring system.

The property distributions in Figure 3 highlight that compounds from clinical trials and drugs tend to have quite similar 1d property distributions. We can use these distribution plots when predicting future ring systems to filter out ring systems where the heteroatom ratio is significantly outside of the typical distribution plots for drugs and clinical trials. For example, it is unlikely for a ring system to contain more than 20% sulfur atoms. Likewise, there is no bias toward the different percentages of sp^3^ centers. Furthermore, the most prevalent ring system size for both drugs and clinical trials is bicycles.

## Future of Ring Systems in Drugs and Clinical Trials

Using our database of clinical trials and drug ring systems we can make a prediction of future clinical trial ring systems and possible new ring systems that will make it as drugs. By simple visual inspection it became apparent that many of the ring systems in clinical trials are very small changes on known drug rings, which could reflect the constraints of biological space and how we choose to effectively navigate chemical patent space or for synthetic tractability reasons. We explored this observation systematically by first restricting the ring systems to a maximum of five rings per ring system. We then assessed the relationship between the drug ring systems and new ring systems in clinical trials. Our systematic approach changes the drug rings by no more than two atoms (*N* + 2) where a change is a single atom substitution, for example, C to N, or the addition or removal of a new exocyclic double bond. We made these changes for all drug ring systems and then compared the *N* + 2 changes to see how many of the ring systems in clinical trials are covered by this simple change on the drug set. We also implemented valence bond checks and substructure-based stability filters on these sets.

The results for this analysis are given in Table 6, where overall 36% of the new ring systems in clinical trials are single atom changes on ring systems in drugs. However, more importantly, approximately one-half of the new ring systems in clinical trials (47%) are at most two atom changes on ring systems in existing drugs. We can use this information to predict the ring systems that will make it into future drugs by applying the same two atom changes to all ring systems from drugs and clinical trials. From this we derived a set of future clinical trial ring systems. This means that from the 30% of drugs that are Class 2 or above (i.e., contain at least one novel ring system), approximately one-half of the molecules could be predicted to have the novel ring systems from our future clinical trial set. However, to ensure that these molecules are reasonable, we wanted to compare these ring systems to those that have been synthesized or reported in the literature, and so we required a full database of ring systems for the currently available chemical space.

**Table 6:** Analysis of New Ring Systems and Overlap with Drug Ring Systems after Applying One or Two Atom Changes^a^

status	no. of new ring systems	single atom change overlap with drug ring systems	two atom changes overlap with drug ring systems
Phase 1	71	31 (44%)	35 (49%)
Phase 2	129	36 (28%)	55 (43%)
Phase 3	76	31 (41%)	40 (53%)
all phases	276	98 (36%)	130 (47%)

a For ring systems containing less than 6 rings.

## Available Chemical Scaffold Space: RINGO Database

To fully understand the magnitude of chemical space associated with ring systems (or chemical scaffolds), we created a database of ring systems from real compounds that are either commercially available and/or synthesized and reported in the literature or patents. Our internal RINGO database of all available ring systems uses ring systems from a data set of approximately 2.24 billion unique molecules taken from commercial, literature, and academic sources including ChEMBL,^41^ eMolecules,^54^ Enamine Real,^22^ SureChEMBL,^55^ etc. We have not included the virtual databases from GDB^23,56^ (Enamine Real is included as each compound has an associated synthetic route and corresponding reagents). The full molecules are first preprocessed and charges and tautomers are calculated for all 2.24 billion molecules using the tautomer and protonation plugins within Chemaxon.^57^ This is an important step since the fragmentation rules for molecules require sp^3^ and sp^2^ centers to be correctly defined, for example, keto vs enol forms. These molecules are then recursively fragmented into the individual ring systems retaining the growth vectors from the original molecule and the frequency for each ring system from the 2.24 billion molecules. From this computation we derived our RINGO database of 458 748 ring systems which covers the known chemical ring space. It is worth noting that 167 668 of the ring systems in RINGO have only been recorded in one compound across our public and commercial sources. Over 80% of these singletons were predominately from compounds in the public database PubChem or the patent database SureChEMBL. Ring systems with high frequencies in RINGO are likely to be synthetically tractable, and while the reverse statement will not always be true, ring system frequency is another factor by which we can filter scaffold space.

We then cross referenced the future clinical trial ring systems against our internal RINGO database of over 458 748 ring systems. This allows us to check whether these ring systems of two atom changes have ever been included in a synthesized molecule and are reported in the data set over 100 times. We also used our previous analysis of the content of drug rings to reduce this set further by applying cut offs for the maximum number of nitrogens, oxygens, and sulfurs in drug ring systems. This gives a “future clinical trials” set of 3902 ring systems out of a possible 458 748. We therefore reduced the set of ring systems to a focused set of around 0.85% of the available chemical ring systems. This can be compared with the drug ring systems which are a privileged set of approximately 0.082% of the chemical scaffolds available.

**Figure 4:**
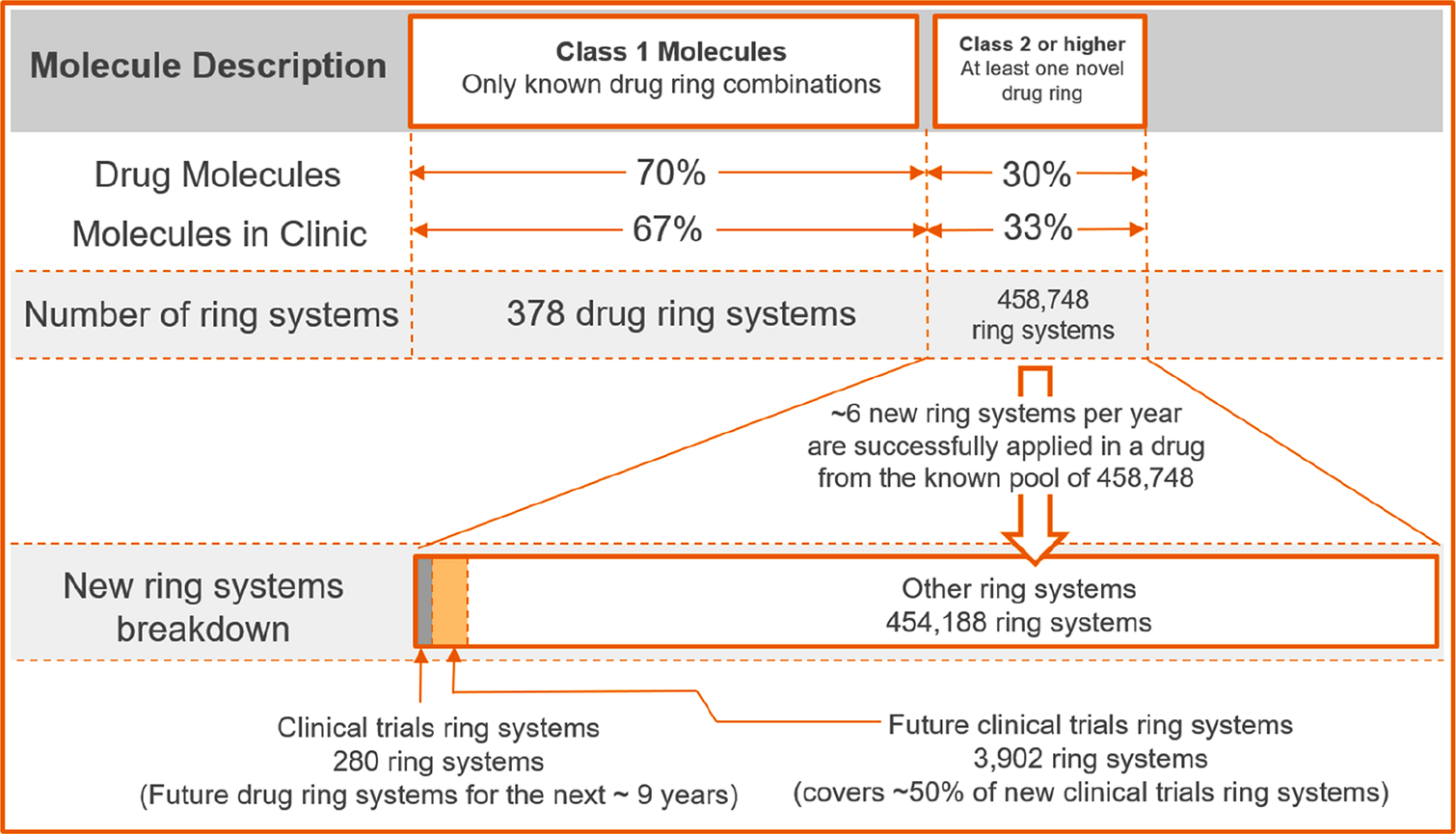
Summary of molecular ring systems (scaffolds) in drugs, clinical trials, reported/synthesized chemical space, and predicted future clinical trial ring systems.

We can thus predict 85% of all new drugs (70% of drugs being Class 1 and one-half of the remaining 30% of Class 2 and above) will come from a combination of ring systems from drugs (378), clinical trials (280), and future clinical trials (3902), which is approximately 1% of the currently reported ring systems. This analysis thus has practical application to library design and is summarized in Figure 4.

## Analysis of Growth Vectors

An additional layer of complexity to add to the previously defined classification system is not only the content of the ring systems but also how they are combined and the associated linking vectors, i.e., the points of attachment from the ring systems. There are subsets of Class 1, Class 2, and Class 3 where the known ring systems use either (i) only the known vectors for linking and growth or (ii) a combination of novel and known vectors or (iii) novel vectors only.

From our database of clinical trials and drug molecules we recorded all unique growth vector combinations for each ring system when we generate the frameworks. We collated this set and compared the clinical growth vectors with those of drugs (see Table 7), and in both cases, we recorded the enantiomeric form of each growth vector. There is approximately a 40% overlap of total ring space of clinical trial ring space with drug ring space, but if the growth vector combinations are included in the comparison then the overlap drops by about 15%, implying that for the drug rings being reused in clinical trials one-third of those utilize a different set of growth vectors which would achieve additional novelty. This suggests that around three-quarters of the drug rings that are used in clinical trials are not only the same rings but also the same points of attachment.

**Table 7:** Summary of Growth Vectors Used in Ring Systems from Molecules in Drugs and Clinical Trials

status	total no. of unique rings	rings from drugs	no. of unique rings and vector combinations	overlap of vectors and ring systems with drug vectors
Phase 1	191	101 (53%)	377	129 (34%)
Phase 2	278	128 (46%)	583	177 (30%)
Phase 3	169	91 (54%)	290	30 (37%)
all phases	450	170 (38%)	1003	239 (24%)
drugs	378		909	

Another area of interest is the total number of growth vectors used per ring system within drugs and clinical trials. The number of vectors per scaffold can be used to guide how molecules can be assembled along with a suggestion for the number of growth vectors that are typically used for new ring systems.

**Figure 5:**
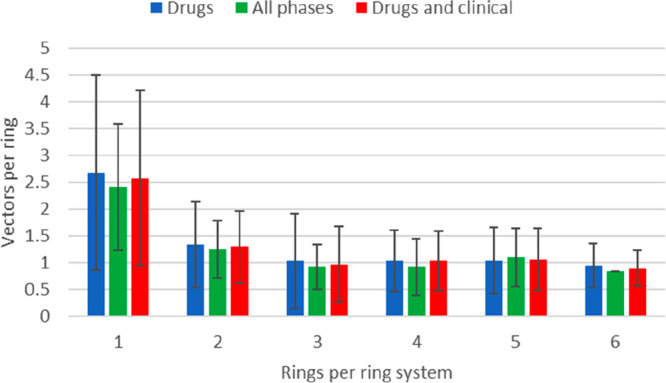
Average number of vectors used per ring vs number of rings per ring system for sets of molecules from drugs and clinical trials. Error bars are the standard deviation of each distribution.

The average growth vector per ring per ring system was determined across “drugs”, “all phases”, and “drugs and clinical” sets (Figure 5). One key observation was that the number of growth vectors per ring was disproportionately higher for monocycles (around 2.5). It is unclear whether the preceding observation just reflected the known chemistry and available synthetic handles on certain monocycles, or an increase in complexity in the rings is often balanced by a decrease in complexity for substitution patterns, or a more physical justification exists (e.g., ortho substitution in aromatic rings to influence rotamer populations or specific protein target interactions based on structure-based design or to prevent a metabolic process). For bicycles and above, our analysis suggested that ring systems in drugs and clinical trials usually have around 1 vector per ring, e.g., a bicycle ring system typically has 2 vectors and a tricycle has 3 vectors.

## Combining Ring Systems and Networks

We have shown that current drug and clinical trial ring systems have a combined total of 678 ring systems out of a possible 458 748 ring systems in available and reported chemical space. The potential number of combinations of these ring systems is huge as we have previously demonstrated by just combining two drug ring systems. A key question when optimizing compounds or indeed designing a library is which pool of ring systems should you pick from to maximize the probability of success while enabling a patent position. Furthermore, this question highlights the importance of the underlying ring systems as if they do not bind to the target or they have an intrinsic liability, i.e., are not productive, then the combinations of rings may also be nonproductive but on a much larger scale.

In this section, we investigated how ring systems are combined to form molecules from drugs or clinical trials. To analyze how ring systems are combined, graph theory was used whereby a series of graph networks were built for each clinical phase, a combined clinical set, and drugs. Each node in the network graph represented a ring system, and if two ring systems were in the same molecule then they were directly connected in the graph. These network diagrams can be used to identify patterns in how ring systems are assembled to form full compounds that are present in the clinic or drugs. This can subsequently be used to bias the design of virtual or screening libraries to clinically relevant chemical space. Similar graph networks are used in social network analysis, and a common way to derive patterns across such complex networks is to cluster the network into sets of nodes (i.e., ring systems) that are densely connected. In this work, the goal of clustering each network was to identify groups of ring systems that frequently occurred together in drugs and/or clinical trials. All networks in this paper were built and then clustered with the Girvan–Newman algorithm^58^ within NetworkX^59^ and then visualized with Cytoscape.^60^ The largest cluster in each network was positioned at the top left of each diagram.

Common statistics for each network are outlined in Table 8, including the graph density and the isolated fraction. The graph density is the fraction of connections in the network compared to whether all nodes were connected, and the isolated fraction is the fraction of nodes that are not connected to anything, i.e., the fraction of molecules where the ring system is not connected to any other ring systems.

**Table 8:** Network Statistics for the Compounds in Each Clinical Phase (Phases 1–3), Combined Clinical Set, and Drug Compounds

status	no. of nodes	no. of edges	isolated fraction	density (×10^–2^)
Phase 1	191	452	0.08	2.45
Phase 2	278	710	0.15	1.83
Phase 3	169	320	0.15	2.22
all phases	450	1184	0.14	1.17
drugs	377	590	0.26	0.83

The clustering of nodes within Phases 1–3 was quite similar, but the densities varied a lot. The density of the drug network was much smaller than that of the combined clinical set and had double the fraction of isolated nodes. It seems that the ring systems in drugs are more sparsely connected than their clinical trial counterparts. This implies greater complexity in connections for clinical trial rings.

**Figure 6:**
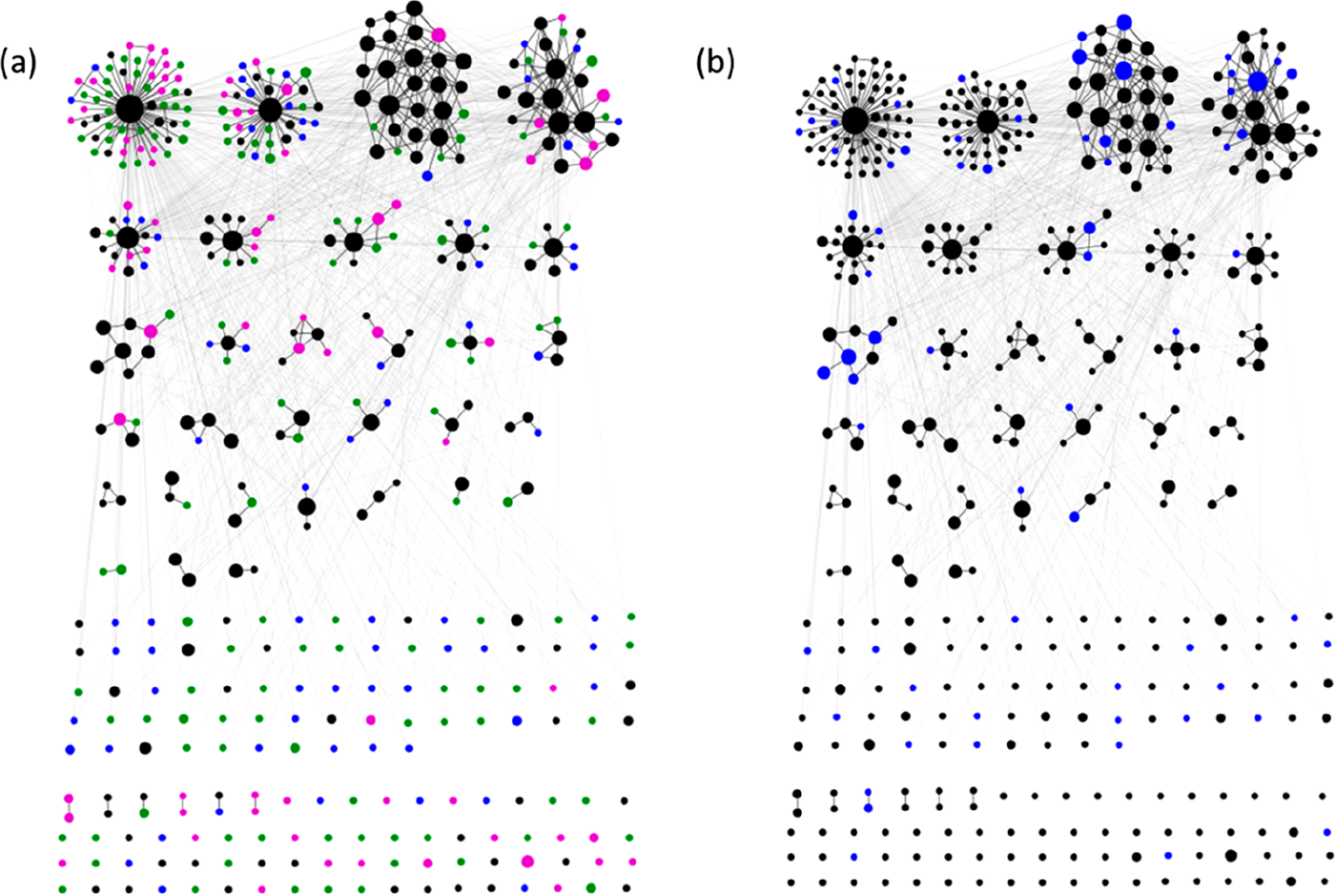
Network diagram showing how ring systems are connected in (a) all compounds in clinical trials. Color of the node represents the highest phase that each ring system can be found. Key: Phase 1 = blue, Phase 2 = green, Phase 3 = purple; drugs = black. (b) Network diagram depicting ring systems in clinical trials that are commonly found in kinase inhibitors (blue nodes); remaining black nodes are all other ring systems present in clinical trials. Central node of the top left cluster (largest cluster) in each subfigure represents benzene.

Next, the property space of the three largest clusters in the drug network (Figure S1) were compared to the overall scaffold space. The property distributions (see Figure S2) for ring count, the number of nitrogens, oxygens, sulfurs, heteroatoms, and the number of sp^3^ centers were calculated for the three largest clusters in the drugs network. There were not many significant differences between the properties of each cluster. Most notably, one cluster (cluster 3) was very deficient in oxygen atoms and monocycles, which could suggest that ring systems with high oxygen contents are not often paired with other ring systems. The property distributions of the three largest clusters in the clinical trial network (Figure 6a) were then determined (Figure S3). There were a few large differences between clusters in clinical trials and drugs: (1) a lower proportion of monocycles in clinical trials and 2) a greater proportion of nitrogen atoms in the clinical ring systems.

One topic of interest was to determine how target-specific ring systems were distributed across the scaffold network. For the sake of simplicity, we focused on the distribution of “kinase-specific” ring systems within the clinical network. Here, kinase-specific referred to any ring system for which over one-half of the compounds it appeared in were kinase inhibitors. It can be seen in Figure 6b that the kinase-specific scaffolds were distributed across the entire network. In general, one kinase-specific ring system would be connected to a popular nonspecific kinase scaffold, e.g., benzene (top left cluster). However, there are a few small clusters in which kinase-specific ring systems are connected to each other. This network clearly identifies privileged ring system pairs that usually appear together in kinase inhibitors. These findings can be used to guide the design of target-specific virtual libraries.

Graph topology measurements, such as how well connected each node is (degree centrality), can be used to identify key ring systems in the drug network (Figure S1). The overlap between the top 10, 20, and 50 scaffolds by frequency and degree centrality was 80%, 75%, and 68%, respectively. These results show that ordering nodes by graph topology measurements has some correlation to ordering by frequency, but these two approaches do not give identical results. For the sake of comparison, the top 10 ring systems by degree centrality are shown in Table 9. The frequency of a ring system in drug compounds gives an idea of how “privileged” a particular scaffold is with respect to “drug space”. However, a ring system could be frequently occurring in drugs but has only been combined with a limited number of ring systems. It could be argued that the design of a general hit ID library should be centered around scaffolds that frequently occur in drugs and those that have appeared in a variety of contexts. The use of network graphs allows for the simple identification of scaffold “hubs” that are frequently occurring and have been reported in a diverse range of compounds. There are a few other graph metrics of potential interest to library design: (1) prioritize scaffolds by how well connected the scaffold and its nearest neighbors are (eigenvalue centrality) or (2) identify scaffolds that connect different clusters (betweenness centrality).

**Table 9:**
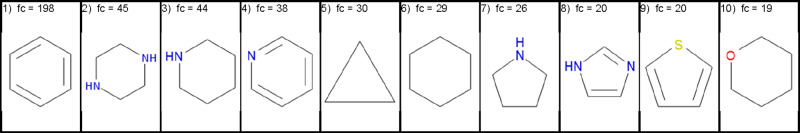
Top 10 Most Frequently Connected Ring Systems from Small Molecule Drugs Listed in the FDA Orange Book before January 2020 Sorted by Descending Frequency of Connections (fc) and Then Ascending Molecular Weight

The potential practical uses of these graphs fall into a few camps: library visualization, library generation, and compound design. The main utility of these graphs is likely for library visualization and interactive analysis. The graphs provide a visual way to determine how central each ring system is to a compound collection, not just by a simple count but by how many other scaffolds it is connected to and thus how integral each ring system is to the chemical diversity of the compound library. Furthermore, these graphs show how different regions of scaffold space are connected and the density of such connections.

## Conclusion

In this work, an in-depth analysis of the scaffold chemical space of compounds in clinical trials has been carried out, and the results have been compared to ring systems in FDA drugs. It was found that around 70% of all clinical trial compounds only contain ring systems that are present in drugs, and we introduced a new classification system for these molecules based on the ring system origins, i.e., Class 1. This result mirrored findings from previous work^15^ in which 70% of all newly released drugs were shown to only contain rings already in drugs. While we may have expected higher novelty in clinical trials when using our classification of molecules through the origin of the ring systems, this was not seen. However, when considering the complete set of ring systems used across all molecules in clinical trials there is a different conclusion in that the overall pool of new ring systems in clinical trials is greater than those ring systems from drugs; therefore, we are introducing more new ring systems in clinical trials. However, this is balanced by more frequent use of known drug ring systems compared with the new ring systems along with different growth vectors and combinations. One area we have not explored in this work is what ring systems are present in compounds that failed in the clinic. While failures in the clinic are of great interest to the field, the data presents a few issues that prohibit drawing meaningful conclusions. Compounds do not always fail at the clinic for scientific reasons and the reason for failure is often not included in clinical databases. Thus, any trends we could draw from failed compounds and the derived scaffolds are not necessarily a direct result of any underlying chemical issue.

It was noted that many novel clinical ring systems were closely related to existing ring systems in drugs. To test this hypothesis, up to two atom changes were performed on all drug rings and the enumerated rings matched to novel ring systems. It was found that around 50% of novel ring systems in clinical trials were within two atom changes of an existing drug ring system.

We carried out one of the largest recursive fragmentation protocols to date on over 2.24 billion compounds that cover all available public and commercial compounds. This “real” chemical space contained over 450 000 unique ring systems (named RINGO database). This data set provides an estimation for all of the rings that are available in synthesizable chemical space, where previous work in the literature has focused on virtual space^38,44^ or bioactive ring space.^44,46^ This data set builds on the work of others^44,46^ to generate drug-like rings via virtual enumeration. Given our earlier observations that around 50% of future clinical trials scaffolds will be within two atom changes of rings in drugs or clinical trials, from 458 748 ring systems, 3902 ring systems were prioritized as future clinical trial scaffolds using not only the two atom change methodology but also heteroatom ratios derived from drugs and prevalence in public and commercial libraries. Using these simple ligand-based rules, we predicted around 1% of the “real” scaffold chemical space will encompass the ring systems used in 85% of new drugs. Moreover, we would highly recommend that the ratios of heteroatoms in ring systems and simple atom changes be used to help prioritize new ring systems that fall outside of the current analysis.

Several analyses were performed to compare growth vectors in drugs and clinical trials and how compounds were built up. There was a 40% overlap between the rings in clinical trials and drugs, but if the growth vector combinations were included in the comparison then the overlap dropped by about 10%. This implied that around one-quarter of all drug ring systems in clinical trials explored novel growth vector combinations. To analyze how compounds were built up in drugs or clinical trials, a graph was built for each collection in which scaffolds that appeared in the same compound were connected by an edge. This analysis showed that, on average, ring systems in clinical trials had been combined with a much wider variety of scaffolds and were half as likely to have never been combined with another unique scaffold. These observations suggested that a greater variety of vector and scaffold combinations are used in clinical trials compared to drugs. This could be symptomatic of the introduction of more structure-based methods and modern synthetic routes in newer compounds found in clinical trials. The number of vectors per ring system as a function of ring systems remained the same in drug and clinical trials. It was noted that ring systems with more than one ring had an average of one growth vector per ring. We believe these observations on vector count per ring system are useful in focusing the directions for not only the optimization of molecules but also the number of vectors used during synthesis of novel ring systems in molecular libraries.

Over the course of the work guidance on what clinically relevant clinical space is most likely to look like has been provided. The authors believe that the analysis described here will provide value through efficiently directed synthesis of clinical candidate molecules which feature fewer liabilities, reducing unknown risk in drug discovery.

## Supporting Information Available

A3 PDF posters and molecule structures in smiles file format for the ring systems in drugs and clinical trials sorted by frequency and then molecular weight. This material is available free of charge via the Internet at https://doi.org/10.5281/zenodo.6556751 (DOCX)

## Abbreviations Used

1D: 1-dimensional
2D: 2-dimensional
3D: 3-dimensional
BM: Bemis–Murcko
CSK: cyclic skeleton
ESO: European Southern Observatory
FDA: Food and Drug Administration
MMPA: Match Molecular Pairs Analysis
NASA: The National Aeronautics and Space Administration
PCA: principal component analysis
PSA: polar surface area
QSAR: quantitative structure–activity relationships
Ro5: rule of 5
SAR: structure–activity relationships
SAVI: Synthetically Accessible Virtual Inventory
