# Supplementary material for: Rings in Clinical Trials and Drugs: Present and Future
Source: J Med Chem. 2022 Jun 22;65(13):8699–712. doi: 10.1021/acs.jmedchem.2c00473 (PMC9289879; doi:10.1021/acs.jmedchem.2c00473)
Supplement: Supplementary file 1 — jm2c00473_si_001.docx [file jm2c00473_si_001.docx]

**SUPPORTING INFORMATION**

**Rings in Clinical Trials and Drugs: Present and Future**

Jonathan Shearer†, Jose L. Castro†, Alastair D.G. Lawson†, Malcolm MacCoss‡, Richard D. Taylor*†

†UCB, 216 Bath Road, Slough, SL1 3WE, UK

‡Bohicket Pharma Consulting LLC, 2556 Seabrook Island Road, Seabrook Island, SC 29455, USA

*To whom correspondence should be addressed.

E-mail: rich.taylor@ucb.com

**Table of Contents**

**Figure S1.** Network diagrams showing how ring systems are connected in drug compounds ………S2

**Figure S2.** Histograms of ring system property distributions within the largest three clusters of the drug network diagram..………………………………………………………………………………..S3

**Figure S3.** Histograms of ring system property distributions within the largest three clusters of the clinical trials network diagram...……………………………………………………………………...S4


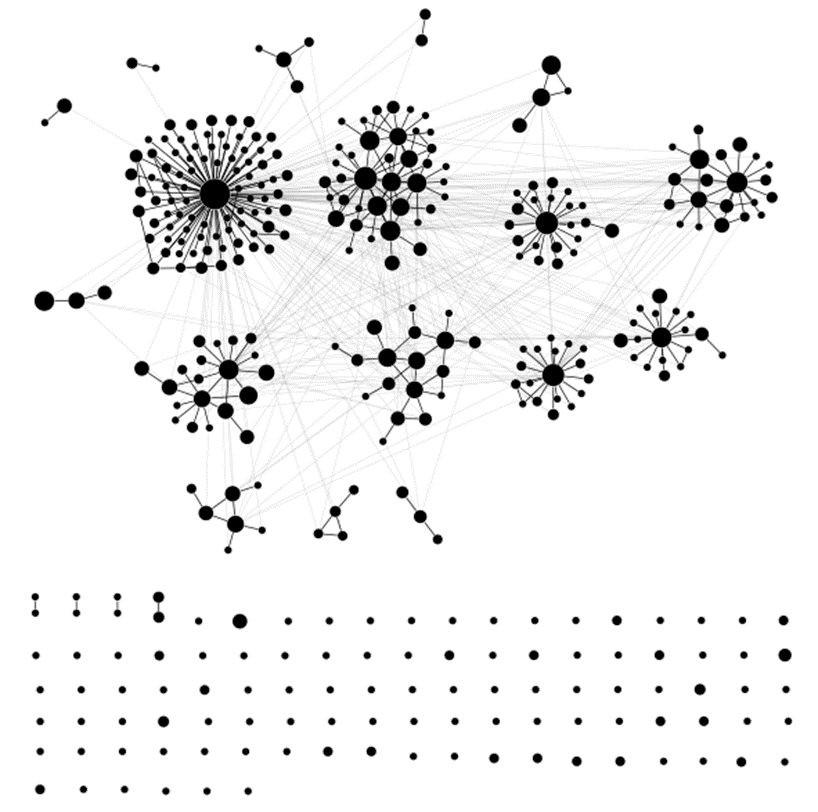


**Figure S1.** Network diagrams showing how ring systems are connected in drug compounds. The central node of the top left cluster (the largest cluster) in each subfigure represented benzene.

**
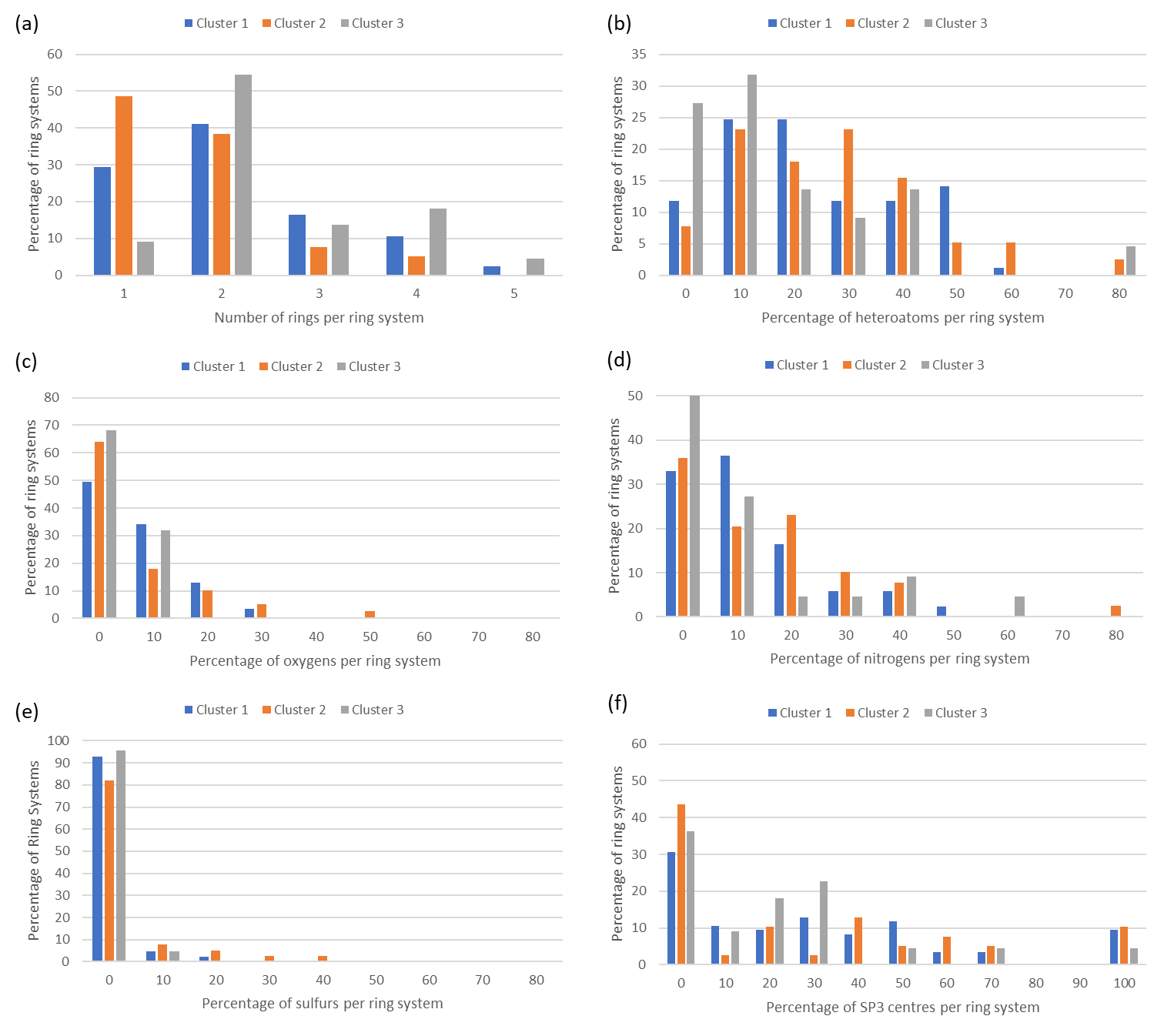
**

**Figure S2.** Histograms of ring system property distributions within the largest three clusters of the drug network diagram: (a) number of rings per ring system, (b) percentage of heteroatoms per ring system, (c) percentage of nitrogens per ring system, (d) percentage of oxygens per ring system, (e) percentage of sulfurs per ring system, (f) percentage of sp^3^ centers per ring system.


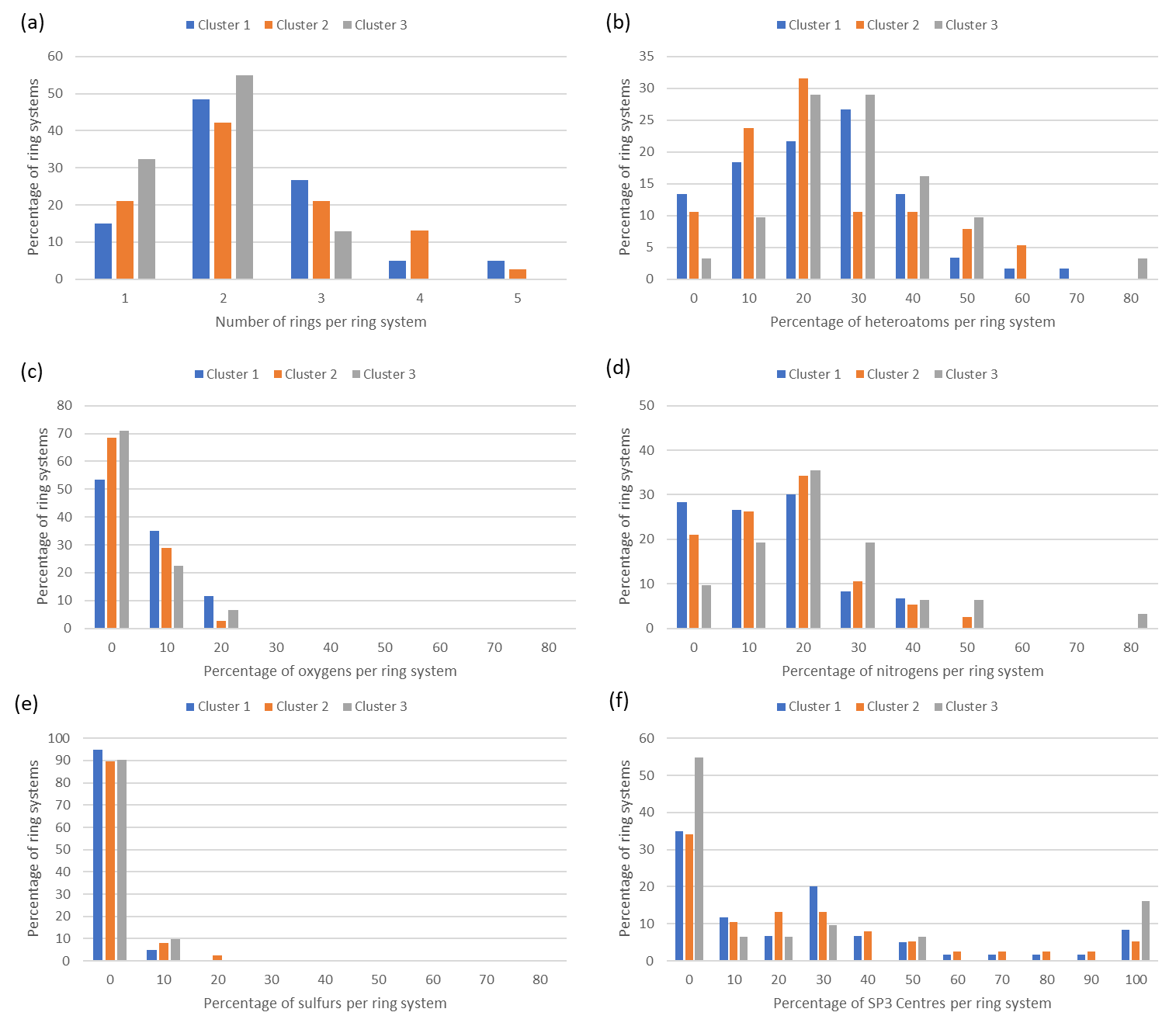


**Figure S3.** Histograms of ring system property distributions within the largest three clusters of the clinical trials network diagram: (a) number of rings per ring system, (b) percentage of heteroatoms per ring system, (c) percentage of nitrogens per ring system, (d) percentage of oxygens per ring system, (e) percentage of sulfurs per ring system, (f) percentage of sp^3^ centers per ring system.
